# Supplementary material for: The rs1883832 Polymorphism (CD40-1C>T) Affects the Intensity of IgA Responses after BNT162b2 Vaccination
Source: Int J Mol Sci. 2022 Nov 14;23(22):14056. doi: 10.3390/ijms232214056 (PMC9697403; doi:10.3390/ijms232214056)
Supplement: Supplementary file 1 [file ijms-23-14056-s001.zip › ijms-1951594-supplementary.pdf]

Table S1. Overview of demographic data, medical history, and adverse effects after vaccination of the enrolled individuals of the study

	Total (n 476)	BNT162b2 (BioNTech) (n 342)	Adenovirus-based vector vaccines		
			Total (n 134)	ChAdOx1 nCoV-19 (AstraZeneca, n 67)	Ad.26.COV2.S (Janssen/J&J, n 67)
Male/female	216/260	136/206	54/80	28/39	26/41
Age (median, range)	55.0, 20.0-105.0	57.0 (27.0-105.0)	49.0 (20.0-84.0)	64.0 (33.0-84.0)	47.0 (20.0-74.0)
**Medical history (n, %)**					
Hypertension	145, 30.5	109, 31.9	36, 26.9	30, 44.8	6, 9.0
Diabetes mellitus	56, 11.8	44, 12.9	12, 9.0	11, 16.4	1, 1.5
Chronic heart disease ^	56, 11.8	47, 13.7	9, 6.7	6, 9.0	3, 4.5
Chronic respiratory diseases *	17, 3.6	15, 4.4	2, 1.5	1, 1.5	1, 1.5
Stroke and transient ischemic attacks	20, 4.2	19, 5.6	1, 0.7	1, 1.5	0, 0.0
Thrombotic (arterial or venous) attacks	8, 1.7	6, 1.8	2, 1.5	0, 0.0	2, 3.0
Chronic liver disease #	4, 0.8	3, 0.9	1, 0.7	1, 1.5	0, 0.0
Chronic kidney disease ^^	5, 1.1	5, 1.5	0, 0.0	0, 0.0	0, 0.0
Thyroid disease **	47, 9.9	36, 10.5	11, 8.2	6, 9.0	5, 7.5
Autoimmunity ##	28, 5.9	26, 7.6	2, 1.5	2, 3.0	0, 0.0
Cancer ^^^	12, 2.5	10, 2.9	2, 1.5	1, 1.5	1, 1.5
Psychiatric diseases***	103, 21.3	100, 29.2	3, 2.2	1, 1.5	2, 3.0
Others ###	87, 18.3	74, 21.6	13, 9.7	10, 14.9	3, 4.5
COVID-19 before vaccination	52, 10.9	40, 11.7	12, 8.9	0, 0.0	12, 17.9
COVID-19 after vaccination	34, 7.1	9, 2.6	25, 18.7	10, 14.9	15, 22.4
**Adverse effects (n, %)**					
Local (available data in 380 participants)	118, 31.1	85, 34.6	33, 24.6	21, 31.3	12, 17.9
Systemic (fever, myalgias, fatigue, etc)	157, 33.0	93, 27.2	64, 47.8	35, 52.2	29, 43.3

Abbreviations: n, number; %, corresponds to the percentage of individuals of the relative cell

^ Chronic heart disease includes atrial fibrillation, arrhythmias, heart failure, angina and myocardial infarction; * Chronic respiratory disease includes chronic obstructive pulmonary disease, chronic interstitial pulmonary disease, bronchiectasis, and asthma; # Chronic liver disease includes chronic HBV hepatitis, primary biliary cirrhosis, and alcohol-related liver disease; ^^ Chronic kidney disease includes chronic renal insufficiency and hydronephrosis; ** Thyroid disease includes Hashimoto disease with hypothyroidism, hypothyroidism (non-autoimmune), and hyperthyroidism; ##Autoimmunity includes autoimmune and/or autoinflammatory diseases like lupus, rheumatoid arthritis, dermatomyositis, scleroderma, psoriasis and psoriatic arthritis, ancylosing spondylarthritis, uveitis, myasthenia gravis, Crohn disease, and ulcerative colitis; ^^^ Cancer includes patients with a history of gastric, breast, thyroid, or kidney cancer and hematologic malignancies (leukemia, Hodgkin lymphoma, Waldenstrom macroglobulinemia, and multiple myeloma); *** Psychiatric diseases includes patients with insomnia, schizophrenia, depression, bipolar disorders, and psychosis; ### Others includes anemia, neutropenia, pernicious anemia, osteoarthritis, osteoporosis, history of epilepsy, Parkinson’s disease, prostate hyperplasia, hydrocephalus, Down syndrome, and history of kidney

Table S2. Antibody responses after vaccination of the enrolled individuals of the study

	Total (n 476)	BNT162b2 vaccine (Pfizer-BioNTech) (n 342)	Adenovirus-based vector vaccines		
			Total (n 134)	ChAdOx1 nCoV-10 (AstraZeneca, n 67)	Ad.26.COV2.S (Janssen/J&J, n 67)
Day 21					
IgG levels (AU/mL) (median, range)	378.6, 0.0 – 40,000	415.8, 0.0 – 40,000	302.6, 7.1 – 22,994.2	255.4, 7.8 – 33,994.2	415.9, 7.1 – 29,806.1
IgG positivity (cut-off: 50 AU/mL) (n, %)	407, 85.5	281, 82.2	126, 94.0	63, 94.0	63, 94.0
IgA levels (U/mL) (median, range)	8.0, 0.0 – 67.6	8.3, 0.0 – 64.9	7.0, 0.1 – 67.6	5.0, 0.1 – 42.7	9.4, 0.1 – 67.6
IgA positivity (cut-off: 10 U/mL) (n, %)	199, 41.9	148, 43.4	51, 38.1	19, 28.4	32, 47.8
Days 42					
IgG levels (AU/mL) (median, range)	5433.2, 0.2 – 40,000	8584.6, 0.2 – 40,000	323.9, 22.4 – 26,246.5	281.0, 22.4 – 26,246.5	423.1, 29.6 – 25,236.4
IgG positivity (cut-off: 50 AU/mL) (n, %)	465, 97.7	335, 98.0	130, 97.0	65, 97.0	65, 97.0
IgA levels (U/mL) (median, range)	17.9, 0.0 – 66.0	22.0, 0.0 – 64.6	4.1, 0.0 – 66.0	2.7, 0.0 – 42.5	6.5, 0.1 – 66.0
IgA positivity (cut-off: 10 U/mL) (n, %)	327, 68.7	293, 85.7	34, 25.4	11, 16.4	23, 34.3
Days 90					
IgG levels (AU/mL) (median, range)	1712.4, 0.0 – 40,000	2138.2, 0.0 – 40,000	735.2, 43.2 – 40,000	898.1, 91.8 – 25934.9	538.7, 43.2 – 40,000
IgG positivity (cut-off: 50 AU/mL) (n, %)	468, 98.3	335, 98.0	133, 99.3	67, 100.0	66, 98.5
IgA levels (U/mL) (median, range)	7.3, 0.0-57.9	8.0, 0.0 – 57.9	4.6, 0.1 – 57.5	3.6, 0.1 – 41.1	5.2, 0.1 – 57.5
IgA positivity (cut-off: 10 U/mL) (n, %)	178, 37.5	136, 39.9	42, 31.3	17, 25.4	25, 37.3

Table S3. Association of IgA responses after vaccination with rs1883832 polymorphism of the enrolled individuals of the study

Vaccination	rs1883832 polymorphism	No	Median, IQR	p
BNT162b2 mRNA vaccine				
Day 21	TT and TC	149	6.36, 11.74	0.059
	CC	143	8.64, 14.49	
Day 42	TT and TC	149	19.19, 15.17	**0.013**
	CC	143	22.3, 17.25	
Day 90	TT and TC	149	5.70, 7.75	**0.026**
	CC	143	8.11, 9.91	
ChAdOx1 nCoV-10 vaccine				
Day 21	TT and TC	31	4.41, 8.62	0.422
	CC	26	5.57, 11.88	
Day 42	TT and TC	31	1.62, 5.69	**0.036**
	CC	26	4.25, 7.04	
Day 90	TT and TC	31	2.69, 10.06	0.424
	CC	26	5.66, 8.15	
Ad.26.COV2.S COVID-19 vaccine				
Day 21	TT and TC	32	9.41, 11.36	0.591
	CC	25	6.49, 11.87	
Day 42	TT and TC	32	4.11, 8.66	0.639
	CC	25	5.18, 8.83	
Day 90	TT and TC	32	4.47, 8.21	0.727
	CC	25	3.68, 10.64	

Abbreviations: IQR, interquartile range; statistical analysis was performed by Mann-Whitney U -test

Table S4. Multivariable analysis exploring the risk factors affecting the anti-SARS-CoV-2 IgG levels at days 42 and 90 after vaccination

**Parameter**	**Co-efficient, 95% CI**	***p***
**Day 42 after vaccination**		
Sex (female/male)	507.33, -1102 – 2116.69	0.536
Age	-125.12, -168.4 – -81.84	**<0.001**
History of COVID-19 before vaccination	14315.04, 11785.00 – 16845.09	**<0.001**
History of COVID-19 after vaccination	125.34, -3031.08 – 3281.76	0.938
rs1883832 polymorphism (het & hom vs wt)	-1126.56, -2673.99 – 420.88	0.153
Vaccination type (BNT162b2 vs adenovirus-based vector vaccines)	5586.78, 4666.97 – 6506.59	**<0.001**
**Day 90 after vaccination**		
Sex (female/male)	-801.30, -1915.28 – 312.68	0.158
Age	3.92, -26.04 – 33.88	0.797
History of COVID-19 before vaccination	11639.93, 9888.65 – 13391.21	**<0.001**
History of COVID-19 after vaccination	2978.06, 793.21 – 5162.91	**<0.001**
rs1883832 polymorphism (het & hom vs wt)	-625.77, -1696.89 – 445.36	0.252
Vaccination type (BNT162b2 vs adenovirus-based vector vaccines)	982.03, 345.35 – 1618.71	**0.003**

Abbreviations: wt, wild type; het, heterozygous; hom, homozygous.

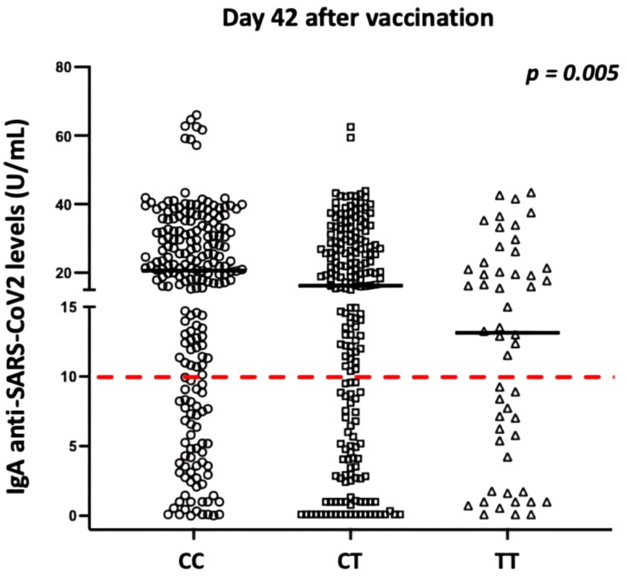

Figure S1. Serum IgA levels according to the status of CD40 rs1883832 polymorphism on day 42 after the first vaccination for all participants of the study. Black lines indicate the median values, and red dotted lines represent the cut-off of positive anti-SARS-CoV-2 IgA (10 U/mL) antibodies. The statistical analysis was performed by Kruskal-Wallis H test.

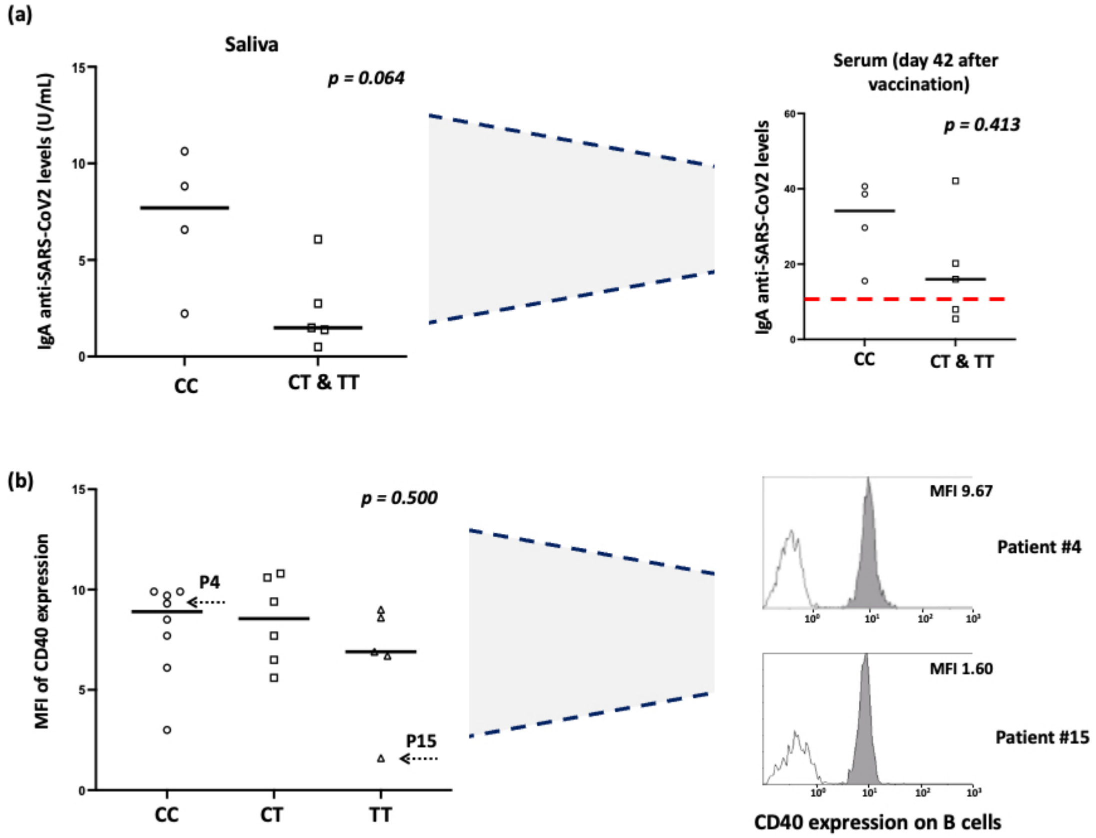

Figure S2. (a) Saliva IgA levels according to the status of CD40 rs1883832 polymorphism (30-45 days after a booster vaccination); the smaller graph indicates the respective serum IgA levels of the same participants on day 42 after the first vaccination. Black lines indicate the median values and the red dotted line represents the cut-off of positive anti-SARS-CoV-2 IgA antibodies (10 U/mL); the statistical anslysis was performed with Mann-Whitney U test. (b) CD40 expression on B cells according to the status of CD40 rs1883832 polymorphism; in the smaller graphs, respective histograms of CD40 expression in two participants with CC and TT gentotype, respectively. Black lines indicate the median values; the sttistical analysis was performed by Kruskal-Wallis H test.
